# Effects of genetic and environmental factors on muscle glycogen content in Japanese Black cattle

**DOI:** 10.1111/asj.12201

**Published:** 2014-04-09

**Authors:** Tomohiko Komatsu, Noriaki Shoji, Kunihiko Saito, Keiichi Suzuki

**Affiliations:** 1 Livestock Experiment Station of Yamagata Integrated Agricultural Research CenterShinjo, Japan; 2 National Livestock Breeding CenterFukushima, Japan; 3 Graduate School of Agricultural Science, Tohoku UniversitySendai, Japan

**Keywords:** environmental effect, genetic parameter, glycogen, Japanese Black cattle, palatability

## Abstract

Monosaccharides such as glucose contribute to the development of meat flavor upon heating via the Maillard reaction; therefore, monosaccharide content is related to beef palatability. Here, we analyzed the effects of genetic and environmental factors on the content of glycogen, one of the precursors of monosaccharides, in the muscles of 958 fattened Japanese Black cattle from Yamagata Prefecture. Analysis of variance showed that muscle glycogen content was affected by the farm and postmortem periods, but not by sex, slaughter age, slaughter month or number of days detained at the slaughter yard. Additionally, consumption of digestible brown rice feed elevated muscle glycogen levels. Glycogen heritability was estimated to be 0.34, and genetic correlations between glycogen and carcass weight (CW) or beef marbling standard (BMS) were weak. The predicted breeding values varied among paternal lines. These results demonstrated that genetic factors might improve muscle glycogen content and therefore beef palatability, but do not influence CW or BMS.

## Introduction

The crude fat content and fatty acid composition of Japanese Black cattle beef have been shown to affect beef palatability (Kobayashi & Shoji 2006; Sakuma *et al*. 2012). A previous study investigating the sensory aroma characteristics of beef demonstrated that crude fat content is positively related with sweet and oily aromas, and that monounsaturated fatty acid concentrations positively affect sweet aroma (Sakuma *et al*. 2012). These traits have been used as markers for the palatability of Japanese Black cattle beef, and the genetic parameters and environmental effects of these traits have been estimated (Oka *et al*. 2002; Inoue *et al*. 2008). On the other hand, water-soluble metabolites, such as glucose and amino acids, contribute to the development of meat flavor upon heating via the Maillard reaction (Maillard 1912). Our previous study showed that monosaccharides such as glucose and fructose are positively related to beef palatability (T. Komatsu *et al*. 2012, unpublished data). Monosaccharide contents in beef are dependent on the muscle glycogen concentration before slaughter (Koutsidis *et al*. 2008) because breakdown of glycogen in muscle cells releases glucose via glycogenolysis during postmortem metabolism. Muscle glycogen content is thought to be affected by various environmental factors. Moreover, muscle glycogen stores are reduced by the stress associated with being held at slaughterhouse facilities with no food (Warriss 1990; Lahucky *et al*. 1998), and degradation of glycogen via postmortem glycolysis occurs up to 48 h after slaughter (Rhoades *et al*. 2005). Furthermore, diet composition (roughage- or cereal grain-based diets) affects muscle glycogen repletion after exercise (Gardner *et al*. 2001).

Therefore, in this study, we investigated the environmental effects of the postmortem period until sample collection, the period of detention with no food in the stockyard before slaughter, and a number of farms where cattle had been raised and fattened with diets of different compositions. The genetic factors affecting muscle glycogen content were then estimated using a mathematical model that factored in the fixed effects of environmental factors. Finally, we evaluated whether muscle glycogen content could be used as a novel marker for meat quality in Japanese Black cattle.

## Materials and Methods

### Animals and carcass evaluation

The Japanese Black cattle analyzed in this study were raised and fattened in Yamagata Prefecture, then slaughtered at a meat center in Yamagata or Yonezawa City between September and December 2011. We chose 958 cattle (170 steers and 788 heifers) from 55 farms. Carcass characteristics of meat located between the sixth and seventh ribs were evaluated using certified graders according to the New Beef Carcass Grading Standards of the Japan Meat Grading Association (JMGA 1988).

### Sample collection

To compare the glycogen concentration in three different muscle types (Trapezius (TM), Longissimus thoracis (LTM) and Biceps femoris (BFM)), samples were collected from 19 Japanese Black steers. Each sample was aged at 4°C for 14 days and then stored at −30°C until analysis. To estimate factors related to glycogen concentration, muscle samples (TM) were obtained from 958 cattle. These samples were collected between 2 and 5 days after slaughter and stored at −30°C until glycogen analysis.

### Glycogen analysis

Muscle glycogen concentrations were analyzed using iodine binding assay according to the method described by Dreiling *et al*. (1987). Approximately 200 mg of raw meat sample was homogenized with 2 mL cold 7% perchloric acid and 2 mL chloroform using Multi-Beads Shocker (Yasui Kikai, Osaka, Japan) for 30 s at 2000 rpm. Samples were subsequently centrifuged at 800 × *g* for 10 min at 4°C. The glycogen-containing upper fraction was collected and used for glycogen measurement. The chloroform fraction was collected, dried and weighed for lipid measurement. The concentration of glycogen was calculated as milligrams per gram of raw meat and lean meat.

### Statistical analysis

For evaluation of differences in glycogen concentration, analysis of variance was performed using the GLM procedures outlined in SAS (SAS Institute Inc., Cary, NC, USA). The correlation between different muscle types was determined using the PROC CORR procedures of SAS. Student's *t*-tests were used to determine differences in muscle glycogen levels.

The mathematical model factored in the fixed effects of sex, slaughter age, slaughter month, farm (the name of the farm where the cattle had been raised and fattened), detained day (period of detention with no food in the stockyard before slaughter) and postmortem day (length of postmortem period until sample collection). Genetic parameters influencing glycogen concentration in the TM, carcass weight (CW), beef marbling standard (BMS), and beef color standard (BCS) were estimated by the following multiple-trait animal model using the VCE6.0 program (Neumaier & Groeneveld 1998): 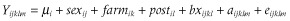
where *Y_ijklm_* is the observation for trait *i*; *μ_i_* is a common constant for trait *i*; *sex_ij_* is the fixed effect of sex *j* for trait *i*; *farm_ik_* is the fixed effect of farm *k* for trait *i*; *post_il_* is the fixed effect of postmortem day *l* for trait *i*; *b* is the coefficient of regression; *x_ijkl_* is a covariate of slaughter age for trait *i*; *a_ijklm_* is the random additive genetic effect of animal *m* for trait *i*; and *e_ijklm_* is the random residual effect for trait *i*. Pedigree information for 3462 animals with data from ancestors was included in this analysis.

## Results and Discussion

### Analysis of glycogen content in different muscle types

**Figure 1:**
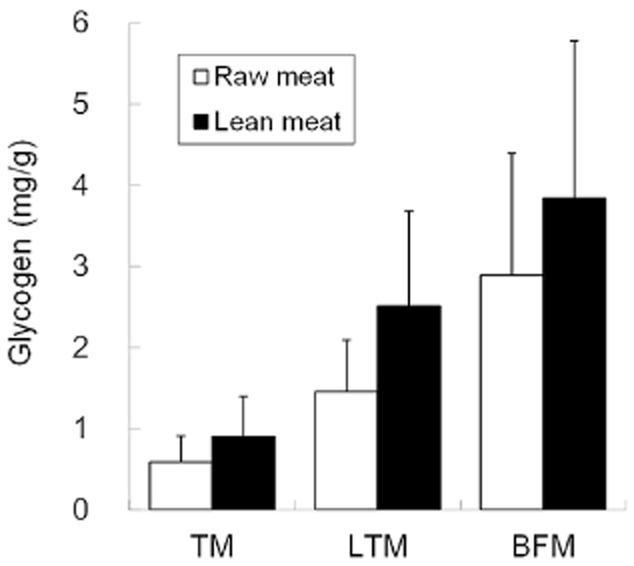
Comparison of glycogen concentration in three different muscles. The mean glycogen concentration in each muscle was calculated and represented as mg/g raw meat (open bar) or lean meat (filled bar). The error bars indicate the standard deviation (*n* = 19). TM, Trapezius muscle; LTM, Longissimus thoracis muscle; BFM, Biceps femoris muscle.

**Figure 2:**
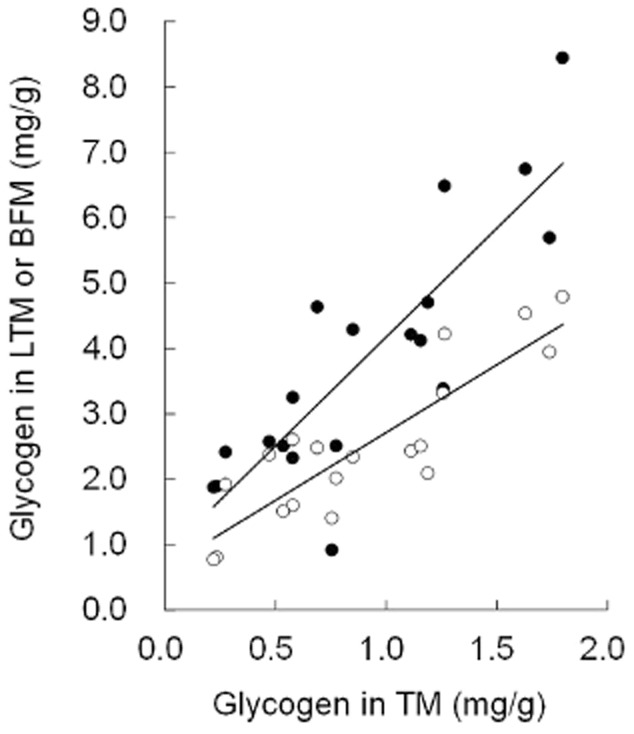
Correlation of glycogen concentration of the Trapezius muscle (TM) with that of the Longissimus thoracis muscle (LTM) or Biceps femoris muscle (BFM) (mg/g lean meat). Each symbol represents the correlation between TM and LTM (○, y = 2.069x + 0.647 (*P* < 0.01, r = 0.87), *n* = 19) or between the TM and BFM (•, y = 3.323x + 0.852 (*P* < 0.01, r = 0.85), *n* = 19).

The glycogen concentration was highest in BFM, lowest in TM and intermediate in LTM. These differences may be related to the muscle fiber-type composition (Fig. 1). BFM is classified as fast twitch or white muscle and is an anaerobic, glycolytic muscle. In contrast, TM is classified as slow twitch or red muscle, which has been shown to be oxidative in nature (Kirchofer *et al*. 2002). Importantly, glycogen content in BFM/LTM was positively correlated with that in TM (Fig. 2). Therefore, we analyzed TM samples to evaluate the effects of genetic and environmental factors on muscle glycogen content.

### Factors affecting glycogen concentration in muscle

Fundamental statistics for carcass traits and analysis traits are shown in Tables 1 and 2. Analysis of variance showed that glycogen concentration was affected by farm and postmortem day until sample collection (Table 3). Rhoades *et al*. (2005) reported that muscle glycogen concentrations decrease by 45% from day 0 to day 4 postmortem. In our study, the mean glycogen concentrations of samples on postmortem days 3 and 4 were lower than those on day 2, by approximately 10% and 32%, respectively (*P* < 0.01). However, there were no significant differences between the mean glycogen concentrations of samples on postmortem days 2 and 5, possibly because the other factors, such as paternal sire and farm, were more significant factors than postmortem day. Analysis of variance showed that sex, slaughter age, slaughter month and number of days detained in the stockyard had a negligible effect on the glycogen concentration compared with other factors, such as farm. The effect of transport stress could not be determined because the cattle analyzed in this study were fattened and slaughtered in the same prefecture, and transport to the slaughterhouse did not require traveling across large distances or long durations. Previous studies have shown that transport stress can be effectively reduced by trimming hoofs (Misumi *et al*. 2001) and by the use of electrolytes (Schaefer *et al*. 1997).

**Table 1:** Fundamental statistics for carcass and analysis traits

Trait	Mean ± SE	Min.	Max.
Carcass trait			
CW (kg)	438.9 ± 57.4	284	631
BMS	7.38 ± 2.07	2	12
BCS	3.92 ± 0.57	2	6
Analysis trait			
Glycogen (mg/g raw meat)	0.63 ± 0.54	0.01	4.14
Glycogen (mg/g lean meat)	0.90 ± 0.75	0.01	7.38

Values are represented as the mean ± standard error (SE). BCS, beef color standard; BMS, beef marbling standard; CW, carcass weight.

**Table 2:** Fundamental statistics for glycogen concentration

Factor	*n*	Mean ± SE
Sex		
Steer	170	0.71 ± 0.04
Heifer	788	0.94 ± 0.03
Slaughter age		
30	187	0.69 ± 0.04
31	74	0.59 ± 0.07
32	245	0.98 ± 0.05
33	253	1.08 ± 0.05
34	96	0.96 ± 0.08
35	63	0.76 ± 0.08
36	40	0.86 ± 0.11
Slaughter month		
September	212	0.99 ± 0.06
October	186	0.93 ± 0.06
November	288	0.80 ± 0.04
December	272	0.92 ± 0.05
Detained day		
0	589	0.95 ± 0.03
1	369	0.83 ± 0.04
Postmortem day		
2	283	1.01 ± 0.05
3	389	0.91 ± 0.04
4	199	0.69 ± 0.04
5	87	0.98 ± 0.10

Values are represented as the mean ± standard error (SE). The concentration of glycogen was calculated as mg/g lean meat. *n*, number of fattened cattle analyzed.

**Table 3:** Analysis of variance for glycogen concentration

Factor	DF	Type III SS	Mean square	F value	Pr > F
Sex	1	0.10	0.10	0.23	0.63
Slaughter age	6	4.95	0.83	1.91	0.08
Slaughter month	3	2.59	0.86	1.99	0.11
Farm	54	101.52	1.88	4.34	< 0.01
Detained day	1	0.02	0.02	0.05	0.82
Postmortem day	3	5.29	1.76	4.07	< 0.01

DF, degrees of freedom; SS, sum of squares.

The estimated fixed effect of farm is shown in Table 4. Although the feed and control systems that were used for the upper and lower groups were similar, some farms used steamed brown rice as feed, which is produced using a unique feed-making process that involves steaming at an elevated temperature and pressure (Yamagata Prefecture & Nogawa Farm 2011). The *in vitro* digestibilities of this feed and conventional feeds were compared using an artificial rumen procedure. After 1 or 8 h, brown rice feed produced more volatile fatty acids (VFAs) than conventional feed, suggesting that brown rice feed was more digestible. The cattle shipped from farm b had been fattened with the addition of brown rice feed during the latter stage of the fattening period. In addition, 30 out of the 87 cattle shipped from farm k were fattened with the addition of this feed. Our data showed that the muscle glycogen levels of cattle fed brown rice (*n* = 30, mean ± standard error: 0.77 ± 0.07 mg/g of lean meat) was significantly higher (*P* < 0.01) than those of cattle not fed brown rice (*n* = 57, 0.39 ± 0.06 mg/g of lean meat). These results suggested that digestible brown rice feed elevates muscle glycogen levels. Dietary glucogenic precursors, such as propylene glycol, are widely used within the dairy industry to treat bovine ketosis (Patton *et al*. 2004). Additionally, Gardner *et al*. (2001) showed that muscle glycogen repletion in cattle fed diets supplemented with glucogenic precursors was marginally better than that of cattle fed with normal rations.

**Table 4:** The estimated fixed effect of farm on muscle glycogen concentration

Farm	*n*	Fixed effect on glycogen
a	8	1.463
b	10	1.052
c	12	1.020
d	10	0.913
e	8	0.893
f	10	0.842
…	…	…
g	12	−0.156
h	6	−0.229
i	11	−0.294
j	218	−0.306
k	87	−0.310
l	6	−0.358

Fifty-five farms are listed in the order of estimated fixed effect on muscle glycogen concentration (the upper six and lower six farms are shown). *n*, number of fattened cattle analyzed.

### Genetic parameters

Glycogen heritability was moderate (*h^2^* = 0.34 ± 0.09), and genetic correlations of glycogen with CW and BMS were weak (−0.29 and −0.21, respectively; Table 5). However, the genetic correlation of glycogen with BCS was 0.69. We were unable to determine whether muscle glycogen level influenced BCS because the BCS that was used for estimation was confined to no. 3 or 4 (21% or 69% of total animals, respectively). Lower muscle glycogen stores at the time of slaughter causes lower lactic acid production after slaughter and higher ultimate meat pH, which may increase the risk of dark cutting (dark, firm and dry) beef (Lawrie & Ledward 2006). In addition, some studies have shown that the residual glycogen concentration can influence the physical and sensory qualities of beef (Immonen *et al*. 2000; Wulf *et al*. 2002). Therefore, it is possible to simultaneously improve both muscle glycogen levels and sensory qualities of beef without affecting other carcass traits, such as CW and BMS.

**Table 5:** Correlations of genetic and phenotypic factors with glycogen and carcass traits

	Glycogen	CW	BMS	BCS
Glycogen	*0.34 *±* 0.09*	−0.29 ± 0.09	−0.21 ± 0.17	0.69 ± 0.18
CW	−0.13	*0.56* ± *0.06*	0.28 ± 0.10	0.02 ± 0.15
BMS	−0.09	0.22	*0.26* ± *0.07*	–0.31 ± 0.28
BCS	−0.17	0.16	−0.42	*0.14* ± *0.05*

Genetic and phenotypic correlation are shown above and below the diagonal, respectively. Heritability estimates (*h^2^*) are on the diagonal (*h^2^* ± standard error). BCS, beef color standard; BMS, beef marbling standard; CW, carcass weight.

Ninety-three sires were documented for the 958 cattle analyzed in this study. In addition, 26 of these were sires for 10–85 cattle each. These 26 major sires are listed in the order of their predicted breeding value of muscle glycogen concentration (Table 6). The sires were classified into four paternal lines: T, K, F and S. The predicted breeding values of sires ranged from −0.416 to 0.376. The sires of the K line, with the exception of sire b (i.e. sires p, r, u, w, x and y), had lower breeding values than the sires of the other lines. K-line sires showed robust growth and had large body frames. Gotoh (2003) reported that the myofiber type composition and diameter differ among breeds. Thus, these characteristics may influence glycogen storage capacity and rapid pre- and post-slaughter glycogen depletion.

**Table 6:** Predicted breeding value of muscle glycogen concentration in the 26 major sires

Sire	*n*	Breeding value of glycogen	Paternal sire	Maternal grandsire	Maternal great grandsire
a	13	0.376	T	T	S
b	21	0.362	K	T	T
c	13	0.256	F	T	F
d	10	0.220	T	S	T
e	38	0.212	T	T	T
f	47	0.210	F	T	T
g	14	0.165	T	S	T
h	36	0.155	T	K	T
i	16	0.150	T	F	T
j	11	0.121	T	S	T
k	11	0.114	T	K	T
l	62	0.049	T	T	T
m	26	0.030	T	T	T
n	15	0.029	F	K	F
o	25	0.016	T	T	F
p	67	−0.002	K	T	T
q	14	−0.035	T	T	S
r	79	−0.062	K	T	T
s	16	−0.085	T	T	K
t	38	−0.117	T	T	T
u	52	−0.203	K	T	T
v	39	−0.235	F	T	T
w	10	−0.256	K	F	F
x	33	−0.302	K	T	K
y	85	−0.356	K	T	K
z	29	−0.416	S	F	F

The 26 major sires are listed in the order of predicted breeding value of muscle glycogen concentration. The sires were classified into the following four paternal lines: T, K, F and S. *n*; number of fattened cattle analyzed.

Overall, our results demonstrated that genetic manipulation approaches could potentially be used to improve muscle glycogen content and subsequently affect beef palatability. Additionally, we found that feeding and pre-slaughter handling influenced the residual glycogen content. Further studies are needed to characterize methods to improve muscle glycogen concentration.
